# Obstetrics

**Published:** 1902-06

**Authors:** W. C. Swayne


### OBSTETRICS.

No complication can be met with in practice which will give rise to more anxiety than the **co-existence of carcinoma of the cervix uteri with pregnancy.**

In such a case the practitioner is confronted with a problem, the solution of which is of great difficulty and which demands the most careful consideration.

The complication of carcinoma uteri with parturition—*i.e.*, the case in which the presence of the growth is not known until labour has set in—is comparatively simple: a living or, at any rate, viable child is in the uterus, and the point for decision is, whether it is possible to deliver *per vias naturales* without destroying the child, or, if it is decided that this is impossible, is it preferable to deliver by Cæsarean section? If it is decided to deliver *per vias naturales*, it must be borne in mind that even after craniotomy this is a proceeding involving grave risk to the mother from the great likelihood of dangerous laceration occurring during delivery; while no further step can be taken towards relieving the condition of the mother without another severe operation. Should the condition be one in which cure of the mother by operation is out of the question from infiltration of the maternal tissues, the child's life is sacrificed without adding either to her comfort or safety. Should the condition be one in which hysterectomy is admissible, delivery by Cæsarean section will not add greatly to the risk and will allow of the simultaneous removal of the affected uterus. Again, in the inoperable case, Cæsarean section will render the child's survival more probable, while the mother's life, already probably only a short one, will not be shortened materially should she survive the operation.

In pregnancy the decision to be arrived at is whether to interrupt pregnancy and then operate on the growth or simultaneously to operate on the growth and terminate pregnancy by removing the gravid uterus either entire or after evacuating it of its contents.

Cumston[1] shows that of 60 cases collected by Chantreuil 25 died or 41 per cent., of 75 collected by West 41 died or 54 per cent. Herman gives the mortality of a series collected by him as 30 per cent., and Cohnstein as 57 per cent. This indicates the danger to the mother. The infantile mortality is also over 50 per cent. The diagnosis has to be viewed from the point of view of, first, the existence of a malignant growth in the cervix of a pregnant woman, and secondly, the existence of early pregnancy in a case of carcinoma. The difficulty in the first place is due to the fact that many of the symptoms of carcinoma are met with in, and may be attributed to, pregnancy; this is especially liable to occur in cases where a malignant growth has occurred shortly after impregnation.

The growth may also be mistaken for the fœtal parts, and induration of a fibrous or cicatricial nature may be mistaken for malignant disease and Cæsarean section unnecessarily undertaken.

Premature labour, either induced or spontaneous, is unfavourable for the child. Herman gives the mortality of the child in cases of premature labour as over 40 per cent. and at term as over 20 per cent.; other observers give a much higher mortality rate for children born at term.

As regards Cæsarean section, out of 40 cases (collected by Bar, Sänger, Leopold, and Cumston) 36 children were born alive, but two died some time after birth.

Cæsarean section is favourable to the survival of the child, but the chances of the mother surviving appear to be remote. In the author's opinion Cæsarean section should be discarded unless it can be immediately followed by a total vaginal or abdominal hysterectomy, and that where this is out of the question a Porro's extirpation is justifiable, in spite of its danger, if the growth has invaded the peri-uterine tissues.

In the consideration of cases in which pregnancy is of less than six months duration, the difficulty seems to be in the choice of operation.

[1] *Am. J. Obst.*, 1902, xlv. 1.

The treatment may consist (1) in the induction of abortion or miscarriage, followed after an interval by the radical removal of the uterus and growth. (2) The removal of the growth by curette. (3) The removal of the growth by amputation of the cervix, either intra- or supra-vaginal. (4) The removal of the uterus entire without evacuation; or (5) its evacuation and removal at the same sitting.

Curetting is useless. Amputation of the cervix in the majority of cases is succeeded by miscarriage; a number of cases recover, but only to sink later from recurrence.

Cumston considers that in the first four months total vaginal hysterectomy is the operation of choice; after this, when the uterus is too large to be removed by this route, vaginal hysterectomy may be done after evacuation, or abdominal total hysterectomy. The author has collected 34 cases of vaginal hysterectomy performed between the second and seventh months of gestation without a single death; while Theilhaber and Bachmann have collected 28 others also without a death. This operation then seems one from which good results may be expected.

Vaginal hysterectomy after evacuation gives good results. Theilhaber has collected 17 cases without a death: the danger appears to be in lacerations of the diseased cervix occurring; involution should not be waited for, as it allows of increase of the growth.

Winter, in 1897, finding himself unable to extract entire a pregnant uterus, divided the posterior wall and thus extracted the fœtus and placenta, after which the uterus was easily removed. As regards the removal by the abdominal route, the mortality of the collected cases appears to reach 30 per cent.

Cumston's views as to the means to be adopted are as follows:—As long as there is any chance of cure, the mother's life alone, and if the case is inoperable the interests of the child alone, should be considered, even though its life may be uncertain.

After the sixth month the author is apparently rather in favour of total abdominal hysterectomy, or the operation of delivering *per vaginam* through the incised posterior uterine wall.

Carstens[1] reports a case in which he performed vaginal hysterectomy for carcinoma in the case of a woman four and a half months pregnant with obliteration of the cervix, the result of a previous operation. As the cervix, or rather its remaining portion, was quite impervious and the evacuation of the uterus as a preliminary was not possible, he pushed a pair of scissors through the spot where he considered the cervical canal to be and opened up the canal; through the opening he extracted the fœtus and placenta. Severe hemorrhage occurred, which was easily checked. In tearing open the cervix the *cul de sac* was opened. The operation was completed in the ordinary way,

[1] *Ibid.*, 74.

clamps being used, and the uterus delivered by means of a myoma screw.

He quotes eleven cases in which vaginal hysterectomy was performed after either spontaneous or artificial evacuation of the ovum at intervals varying from eight to thirty days after the evacuation ; one death occurred.

He also quotes 32 cases in which the operation was performed without interrupting pregnancy, of these three died.

It is shown that pregnancy exercises an unfavourable influence on carcinoma, rendering its growth more rapid. On the other hand, the growth usually becomes more active after delivery than before. The mortality is enormous. Whether treated by incision of the cervix, forceps, scissors, or Cæsarean section, about 50 per cent. of the mothers are lost either at the time or as an immediate consequence of labour.

The author concludes that to allow the continuation of pregnancy in a woman affected with carcinoma is to condemn her to an early death.

Sanderson[1] reports a case of a four and a half months' pregnancy complicated with epithelioma of the cervix. He operated by a combined vaginal and abdominal section, and removed the uterus entire with the fœtus *in situ*. The patient made a good recovery.

He lays down the following propositions: (1) Where pregnancy and operable cancer coexist the life of the mother is alone to be considered. (2) That before the fourth month of pregnancy vaginal hysterectomy is the proper treatment. (3) That after this period the alternatives are (*a*) induction of labour, followed by vaginal hysterectomy, or (*b*) hysterectomy by the combined method.

O'Callaghan and Dardenne[2] report a case in which a hard cauliflower-like growth was detected on both lips of the cervix in a woman, aged 36, who had been married 17 years and never pregnant. Hysterectomy was inadmissible, and therefore as much of the growth and cervix as was possible was removed by the knife and cautery. Eight months later she was examined and found to be about five months pregnant, and rather less than two months later labour commenced and the whole of the lower segment of the uterus was found to be a cancerous mass. Delivery being impossible, Cæsarean section was performed, the child delivered, and the uterus removed through the abdominal incision, a procedure which proved to be one of the greatest difficulty. The patient never rallied, and died from shock within 48 hours.

In such a case as O'Callaghan and Dardenne's there is no alternative to Cæsarean section, but it seems a debateable point whether the removal of the uterus was justifiable in his case. One reason in its favour is the difficulty of drainage through the obstructed lower segment, and secondly, the difficulty of pre-

[1] *Brit. M. J.*, 1901, ii. 1738. [2] *Lancet*, 1902, i. 660.

venting infection of the placental site. One would have supposed that the removal of the uterus by Porro's operation would have obviated these difficulties and saved the necessary difficult dissection through infected tissues. It would appear that the lines of procedure should be as follows:—

*A*. In cases in which the cancer is operable: (1) In the first half of pregnancy, vaginal hysterectomy, either without or after emptying the uterus. (2) In the latter half, or rather with a viable fœtus, Cæsarean section, followed immediately by pan-hysterectomy or removal of the cervix *per vaginam*, delivery and removal of the uterus by either route as may seem best.

*B*. In inoperable cases: (1) In early pregnancy, induced abortion after partial removal of the growth. (2) With a viable fœtus, Cæsarean section or Porro's operation.

WALTER C. SWAYNE.

